# Evaluation of native microalgae from Tunisia using the pulse-amplitude-modulation measurement of chlorophyll fluorescence and a performance study in semi-continuous mode for biofuel production

**DOI:** 10.1186/s13068-019-1461-4

**Published:** 2019-05-11

**Authors:** A. Jebali, F. G. Acién, N. Jiménez-Ruiz, C. Gómez, J. M. Fernández-Sevilla, N. Mhiri, F. Karray, S. Sayadi, E. Molina-Grima

**Affiliations:** 1 Laboratory of Environmental Bioprocesses, Sfax Centre of Biotechnology, University of Sfax, P.O. Box 1177, 3018 Sfax, Tunisia; 2 Department of Chemical Engineering, University of Almería, Carretera Sacramento s/n, 04120 Almería, Spain; 3 Center for Sustainable Development, College of Arts and Sciences, Qatar University, 2713, Doha, Qatar

**Keywords:** Microalgae, Productivity, Biochemical composition, Biofuel, Chlorophyll fluorescence, Photosynthetic parameters

## Abstract

**Background:**

Microalgae are attracting much attention as a promising feedstock for renewable energy production, while simultaneously providing environmental benefits. So far, comparison studies for microalgae selection for this purpose were mainly based on data obtained from batch cultures, where the lipid content and the growth rate were the main selection parameters. The present study evaluates the performance of native microalgae strains in semi-continuous mode, considering the suitability of the algal-derived fatty acid composition and the saponifiable lipid productivity as selection criteria for microalgal fuel production. Evaluation of the photosynthetic performance and the robustness of the selected strain under outdoor conditions was conducted to assess its capability to grow and tolerate harsh environmental growth conditions.

**Results:**

In this study, five native microalgae strains from Tunisia (one freshwater and four marine strains) were isolated and evaluated as potential raw material to produce biofuel. Firstly, molecular identification of the strains was performed. Then, experiments in semi-continuous mode at different dilution rates were carried out. The local microalgae strains were characterized in terms of biomass and lipid productivity, in addition to protein content, and fatty acid profile, content and productivity. The marine strain *Chlorella* sp. showed, at 0.20 1/day dilution rate, lipid and biomass productivities of 35.10 mg/L day and 0.2 g/L day, respectively. Moreover, data from chlorophyll fluorescence measurements demonstrated the robustness of this strain as it tolerated extreme outdoor conditions including high (38 °C) and low (10 °C) temperature, and high irradiance (1600 µmol/m^2^ s).

**Conclusions:**

Selection of native microalgae allows identifying potential strains suitable for use in the production of biofuels. The selected strain *Chlorella* sp. demonstrated adequate performance to be scaled up to outdoor conditions. Although experiments were performed at laboratory conditions, the methodology used in this paper allows a robust evaluation of microalgae strains for potential market applications.

## Background

Amongst the conventional petroleum substitutes, biofuel has attracted the greatest interest over the last century owing to its sustainability and eco-friendly nature. However, alternative first- and second-generation biofuel resources, such as crops and lignocellulosic feedstock, have various drawbacks related to water shortage, deforestation, food crop competition and land use [1, 2]. On the other hand, the judicious exploitation of microalgae, a third-generation biofuel resource, could overcome these problems and make a significant contribution to meeting primary energy demand as well as providing environmental benefits [3–6]. In fact, microalgae have a prominent position as a biofuel feedstock given the range of advantages they offer, including a rapid growth rate, no impact on food security and the ability to grow in poor quality waters and on non-arable lands while, at the same time, converting CO_2_ into carbon-rich lipids [7–9]. Nevertheless, although biofuel production from microalgae is technically possible, it is currently economically unfeasible due to the high cost of biomass production and processing. Additionally, sustainability of biofuel production from freshwater species has been questioned in several reports (http://energyskeptic.com/2015/algae/). Microalgae cultivation in seawater is among the few possible options for biomass production for producing fuels at any significant scale. Even so, freshwater is still needed to compensate evaporative losses and a consequent increase in culture salinity [10].

Extensive studies have been carried out to explore locally isolated microalgae strains from the northern Mediterranean for use in biofuel production [11–15]. However, exploring the potential of microalgae from the south-western Mediterranean is still in its infancy. Tunisia is one of the south-western Mediterranean countries that possess various biota with untapped microalgae diversity along with a large non-arable surface area (totalling almost 40%) as well as suitable weather for microalgae growth − 64,000 GJ/ha of solar radiation a year—making microalgae cultivation for biofuel production theoretically feasible [16]. However, it has been recommended that, prior to large-scale production in a given area, selection of the best performing locally isolated strains should be carried out. This is because the native isolates, which are well adapted to local conditions, exhibit better performance and robustness than those from a strain bank collection [17]. Thus, for microalgae strains screening, current studies mainly focus on the biomass concentration and lipid content, often determined from batch cultures [18–22]. Whereas to adequately select the best candidate to be used for biofuel production, the study should be carried out in continuous operational mode, which is closer to the industrial commodities production standards and the target must be to maximize both the biomass and the saponifiable lipid productivities (i.e., fatty acid methyl esters [FAMEs] productivity) in addition to the suitability of the algal-derived fatty acid composition [23, 24]. However, few studies have investigated these criteria in continuous cultures for selecting the best performing strain and the optimal dilution rate [25–27]. This selection approach would allow determining the best combination of the selected criteria above mentioned.

Whatever the operation mode considered, the evaluation of the performance of microalgae cultures is usually based on biomass growth; the utilization of fast methods such as fluorescence of chlorophylls is usually disregarded. This methodology is useful for studying photosynthesis mechanisms [28–31]. The technique using the Pulse-Amplitude-Modulated fluorometer (PAM) is a sensitive tool for examining the variability of chlorophyll fluorescence and photosynthetic performance in microalgae. It allows not only to measure the photosynthetic activity in a rapid and non-intrusive mode but also to investigate processes such as photoprotection and photoinhibition, the physiological status of the cell subjected to stress as well as to estimate the primary production [32–34]. Consequently, the measurement of chlorophyll fluorescence allows a rapid and real-time determination of the photosynthetic performance of microalgae cultures under various conditions influencing microalgae growth. For instance, temperature and irradiance are the most important variables for controlling microalgae culture growth [35, 36]. Thus, these factors are considered among the main environmental parameters affecting photosynthesis in outdoor microalgae massive production [37].

In this study, the isolation and molecular identification of five native microalgae strains commonly distributed in north-eastern Tunisia were conducted. To select the best performing strain and the optimal culture conditions, experiments were carried out in semi-continuous mode. The biomass productivity, the photosynthetic efficiency and the biochemical composition of the different novel isolates were investigated at various dilution rates, to select the optimal one. In addition, the robustness and the suitability of the selected strain for outdoor pilot scale cultivation were evaluated. An assessment of the photosynthetic capacity and parameters of this microalgae strain towards outdoor environmental conditions, namely high and low temperature and high light intensity, was carried out by chlorophyll-fluorescence analysis using the Pulse-Amplitude-Modulated fluorometer (PAM).

## Methods

### Isolation of microalgae strains

Five different strains from north-eastern Tunisia were isolated. Strain WT6 was isolated from a thermal freshwater source; whereas the others, Strain WT1, Strain WT3, Strain WT4 and Strain WT7 were isolated from sea water. The specific location for collecting samples is shown in Table 1. The procedure for isolating these monoalgal strains was as follows: approximately 10 mL of water sample was filtered through a 0.70-µm filter; the cells remaining on the filter were then inoculated in a 250-mL conical flask followed by incubation for enrichment under a continuous light intensity of 80 µmol/m^2^ s at 25 °C with no agitation. In the case of the marine microalgae strains, it was inoculated into a 150 mL f/2 culture medium [38], which was then supplied with silicate at a concentration of 0.01 g/L for the diatoms; or in the case of the freshwater microalgae strain, inoculated with MDM culture medium [39]. After 20–30 days, the cells were purified by successive serial dilutions and then plate streaked in 1.50% agar MDM or f/2 media. Once single colonies had formed, they were picked up and transferred for sequential liquid sub-culturing. Microscopic observations were carried out to verify the purity of the transferred colonies. Morphological identification of the isolated strains was performed, followed by molecular identification (Table 1).

**Table 1:** Sampling site description and characterization of isolated microalgae strains

Algal class	Strain	Habitat	Sampling site	Average cell size (Length, µm × width, µm)	Accession number of sequenced regions:
Bacillariophyceae	*Amphora* sp. WT4	Sea water	36°51′0″ N11°6′0″ E	18.10 × 4.30	18SrRNA:KX109777 *rbc*L:KX109774
Bacillariophyceae	*Nitzschia* sp. WT7	Sea water	36°49′0″ N 10°34′0″ E	15.30 × 3.80	18SrRNA:KX109778 *rbc*L:KX109775
Chlorophyceae	*Scenedesmus*sp.WT6	Fresh water	36°49′0″ N 10°34′0″ E	7.70 × 4.30	18SrRNA:KT267272
Chlorodendrophyceae	*Tetraselmis* sp.WT3	Sea water	37°0′6″ N 10°53′42″ E	11.10 × 8.60	18SrRNA:KX109779 ITS:KX109780
Trebouxiophyceae	*Chlorella* sp. WT1	Sea water	36°58′0″ N 11°4′60″ E	4.10 × 3.90	18SrRNA:KX109776

### DNA extraction, amplification and 18S rRNA, *rbc*L-3P and ITS sequencing

The genomic DNA from Strain WT7, Strain WT4, Strain WT1, Strain WT3 and Strain WT6 was extracted using the FavorPrep Plant genomic DNA extraction mini kit in accordance with the manufacturer’s instructions. The sequence of the 18S rRNA gene (*SSU* rRNA) was obtained by PCR amplification using the primers EukA (5′-AACCTGGTTGATCCTGCCAGT-3′) and EukB (5′-TGATCCTTCTGCAGGTTCACCTAC-3′) [40]. The PCR program for the 18S rRNA gene was as previously described [26]. Additionally, the PCR reaction was performed on the nuclear rDNA spacer sequence (internal transcribed spacers ITS-1 and ITS-2) from the Strain WT3 isolate. The ITS1 (5´-TCCGTAGGTGAACCTTGC GG-3´) and ITS4 (5´-TCCTCCGCTTATTGATATGC-3´) primers were used [41]. The PCR conditions were as follows: 30 cycles of denaturing at 95 °C for 30 s; annealing at 58 °C for 45 s and extension at 72 °C for 1 min; followed by final extension at 72 °C for 10 min. The *rbc*L-3P gene (860 bp) (encoding the large subunit of ribulose-1, 5-bisphosphate carboxylase/oxygenase: RuBisCO) from the Strain WT7 and Strain WT4 isolates was amplified using CfD (5′-CCRTTYATGCGTTGGAGAGA-3′) and DP*rbc*L7 (5′-AARCAACCTTGTGTAAGTCT-3′) primers [42]. The thermal profile for PCR amplification of *rbc*L-3P included an initial denaturation at 94 °C for 2 min, followed by 30 cycles at 94 °C for 20 s, 50 °C for 30 s and 72 °C for 2 min; then a final extension of 72 °C for 10 min. All PCR reactions were performed as previously described [26]. Nucleotide sequences of the *rbc*L gene were translated into amino acid sequences by EMBOSS Transeq (http://www.ebi.ac.uk/emboss/transeq). Target sequences were analyzed using BLAST online (http://www.NCBI.nim.nih.gov/). Multiple alignments were generated with the MUSCLE program [43] and dendrograms were constructed with MEGA program version 6 [44] based on the evolutionary distances that were calculated by the Neighbor-Joining method [45] with the Jukes–Cantor model (18S rRNA and the internal transcribed spacers ITS-1 and ITS-2) and the Poisson model (*rbc*L-3P). Statistical evaluation of the tree topologies was performed by bootstrap analysis with 1000 re-samplings [46]. Sequenced regions of the 18S rRNA gene, the ITS and the *rbc*L gene were deposited in the GenBank database and registered under the accession numbers presented in Table 1.

### Experimental conditions in semi-continuous mode

Firstly, inoculums from the native isolates were prepared at 22 °C, under continuous white light at an intensity of 277 µmol/m^2^ s and continuous aeration in 1-L flasks, where Algal medium (Bionova, Santiago, Spain) was used for the marine strains (the diatom medium was supplied with silicate at a concentration of 0.01 g/L) and MDM medium for the freshwater strain. The inoculums were used to inoculate indoor bubble-column photobioreactors (0.50 m in height, 0.07 m in diameter). The vessels were provided with an air inlet at the bottom (0.50 v/v min) to ensure mixing and dissolved oxygen removal, whereas the valves of the culture harvest and medium inlet were located at the top of the reactor. The culture pH was continuously measured using a pH probe (Crison Instruments, Spain) and maintained within the 7.90–8.00 range by on-demand CO_2_ injection. The temperature was set at 22 °C and kept constant by controlling the room temperature. The illumination, measured by a 4π quantum scalar irradiance sensor (QSL-100, Biospherical Instruments, San Diego, CA, USA), was supplied with six white-light lamps with a maximum irradiance of 1000 µmol/m^2^ s, simulating the circadian cycle. Experiments were conducted in batch mode for 10 days; after that, the cultures were shifted to semi-continuous operation at different dilution rates. Semi-continuous mode was maintained until steady state was reached (washing the reactor volume three times)—data from the last 3 days, for each one of the dilution rates tested, were used.

### Biomass concentration measurements

Samples were withdrawn daily to estimate biomass concentration by optical density at 750 nm (OD 750) using a spectrophotometer (ATI UNICAM UV/Vis Spectrometer UV2, Cambridge, UK). Biomass dry weight measurements were carried out twice a week by centrifuging the microalgae cells for 10 min at 5000 rpm (SIGMA 4–15 Sartorius, Goettingen, Germany). The pellet was washed twice and lyophilized (TELSTAR Cryonos–50, Madrid, Spain).

### Chlorophyll fluorescence measurements

The maximum quantum efficiency of photosystem II, calculated as the Fv/Fm ratio of each microalgae strain, was measured daily (AquaPenAP 100, Photon Systems Instruments, Drasov, The Czech Republic) to monitor the cells’ physiological status; where Fv was the variable fluorescence in dark-adapted microalgae cells and Fm was the maximum fluorescence from the dark-adapted microalgae cells.

The kinetic and photosynthetic parameters of photosystem II (PSII) were estimated by means of the pulse-amplitude-modulation (PAM) measurement of chlorophyll fluorescence using the Junior PAM fluorometer (Junior PAM 2000, Walz, Effeltrich, Germany). Indeed, absorbed light energy results in Chl *a* molecule excitation, which can be de-excited by three different pathways. Excitation energy can be (i) transferred to reaction centers and used in photosynthetic electron transport, photochemistry, (ii) dissipated as heat in the case of excess light energy (closure of reaction centers) and/or (iii) it can be re-emitted as fluorescence [31, 33, 47]. The two mechanisms, photochemistry and thermal dissipation of excitation energy, are known as photochemical quenching (qP) and non-photochemical quenching (NPQ) of chlorophyll fluorescence. Using this technique, the photosynthetic response of whatever microalgae strain exposed to culture conditions prevailing outdoor can be obtained through the generation of light-response curves (RLC) of the various chlorophyll-fluorescence parameters; namely, rETR, alpha, Ik and MQY. The rETR represents the relative maximum Electron Transport Rate through the PSII complex, which is correlated with the microalgae’s overall photosynthetic performance. Alpha is the initial slope of RLC, which reflects the quantum efficiency of the photosynthetic electron transport. Ik is the irradiance that is saturating photosynthesis and MQY is the Maximum Quantum Yield representing the maximum PSII efficiency in the dark-adapted sample [33, 48]. Accordingly, a rapid light curve can be provided from the pulse-amplitude-modulation measurement of chlorophyll fluorescence giving an idea of the photosynthetic activity, the strain’s potential over a Photosynthetically Available Radiation (PAR, 400–700 nm) range and the photosynthetic characteristics (rETR, MQY, Ik and α) [31, 49].

Measurements were performed by immersing the optic fiber in algal culture sample previously incubated for 10 min in darkness. During this measurement, the sample recipient was covered with aluminum foil to avoid interference from the measuring beam on the PAM fluorometer. The initial fluorescence (*F*_0_), the dark-adapted minimal fluorescence flux, was measured with a weak pulsed measuring light at a very low photon fluence rate of 2 µmol/m^2^ s. Then, the maximum fluorescence (*F*_*m*_), the dark-adapted maximal fluorescence flux, was determined by giving a 0.8-s short-saturation high-intensity light pulse of 3000 µmol m^−2^ s^−1^. The ratio of variable to maximal fluorescence (*F*_*v*_/*F*_*m*_) was used as a measure of the maximal photochemical efficiency of photosystem II, where *F*_*v*_ = *F*_*m*_− *F*_0_. This ratio was used to estimate the degree of photoinhibition after irradiation [29]. The time course of fluorescence quenching, *F*, was monitored at different actinic light irradiances during which the fluorescence spike (*F*′_*m*_) was periodically measured by giving pulses of saturation light. The rapid light curve obtained from the chlorophyll-fluorescence measurements consists of three regions: light limited, light saturated and light photoinhibited. The maximal quantum yield (MQY) is considered as the first Y value from the rapid light curve [50].

The slope of the light-limited region is alpha (α) and the rETR (relative Electron Transport Rate) was estimated by the product of the quantum yield (QY) of PSII (*F*′_*m*_− *F*), the photon flux density PFDa (absorbed light) and 0.5, a factor that accounts for energy partitioning between PSII and PSI, as follows:

$$rETR = PFDa \times QY \times 0.5$$

Ik is determined by the equation:

$$Ik = \frac{rETRmax}{\alpha }$$

These characteristic parameters were determined by fitting the light curve using the Platt and Gallegos equation [51]. WinControl software was used to export the data.

Different parameter variations, such as high (38 °C) and low (10 °C) temperature, and high light intensity (1600 µmol/m^2^ s) were applied to the selected microalgae strain for 4 h to assess its tolerance to potential changes in outdoor environmental conditions. Culture samples were maintained at the same cell density for the different measurements through dilution. All measurements were performed in triplicate.

### Biochemical composition and fatty acid profile

At steady state, biomass samples were harvested by centrifugation, washed twice and freeze dried. The protein content was determined according to the modified Lowry method proposed by Lòpez et al. [52]. Briefly, after Lowry and Folin reagents addition, the extract was incubated for 30 min in darkness. Then, the optical density was measured at 750 nm and the protein concentration was determined by a calibration curve using Bovine Serum Albumin (BSA) as standard. Total lipids were quantified as described by Kochert [53] using chloroform–methanol 2:1 (v/v) for the extraction followed by the addition of HCl (0.1 N) and MgCl_2_ (0.5%). Subsequent centrifugation was carried out for lower phase recovery. The lipid content was determined gravimetrically. The fatty acid profile and content were obtained by direct transesterification using hexane, acetyl chloride and methanol as extraction solvents. Nonadecanoic acid (C 19:0) was used as internal standard [54]. Gas chromatography (Agilent Technologies 6890 N Series Gas Chromatograph, Santa Clara, CA, USA) analysis was performed as described by Rodríguez-Ruiz et al. [54]. All analyses were conducted in duplicate.

### Statistical analysis

The Statistical Package for the Social Sciences (SPSS for Windows version 24.0; SPSS Inc., Chicago) was used for the statistical analyses. One-way analysis of variance (ANOVA) and combined analysis of variance were performed using the SPSS software (SPSS, Inc.). The level of significance was expressed as significant at *p* < 0.05 (*) and highly significant at *p* < 0.001 (**). To compare the means between variables, the Duncan test was used.

## Results and discussion

### Phylogenetic analysis

**Fig. 1:**
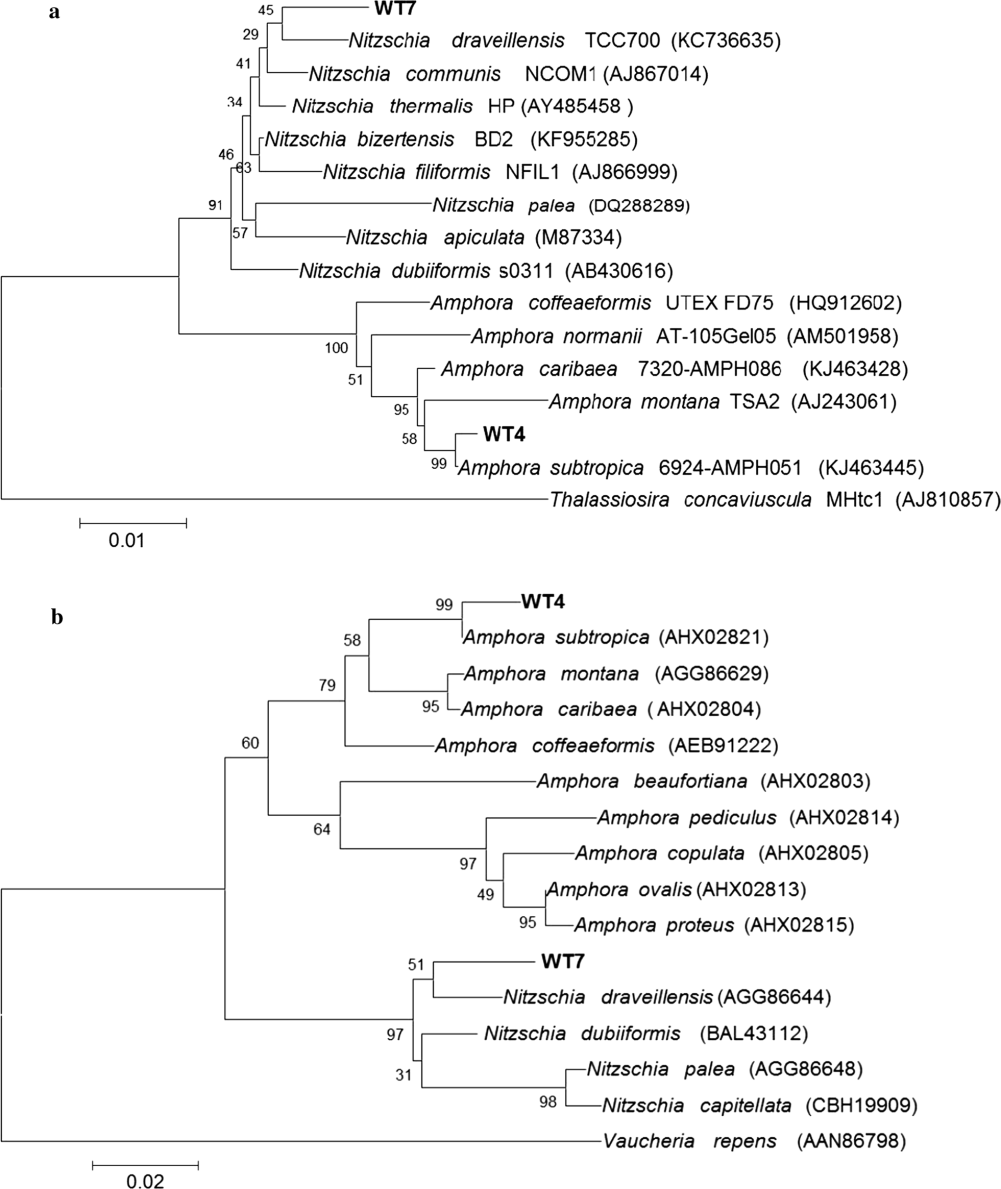
**a** Dendrogram based on the 18S rRNA gene sequence. Bootstrap values are given at the nodes. The scale bar represents the substitution percentage. *Thalassiosira concaviuscula* was used as the outgroup. GenBank accession numbers follow the species name in parentheses. **b** Dendrogram based on the *rbc*L-3P sequence. Bootstrap values are given at the nodes. The scale bar represents the substitution percentage. *Vaucheria repens* was used as the outgroup. GenBank accession numbers follow the species name in parentheses

**Fig. 2:**
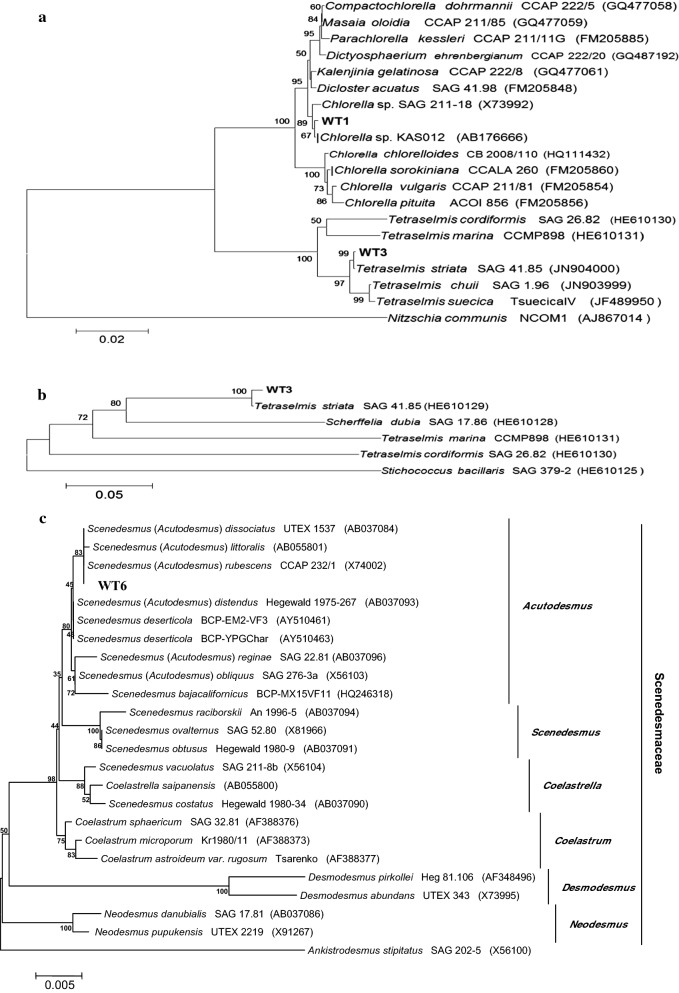
**a** Dendrogram based on the 18S rRNA gene sequence. Bootstrap values are given at the nodes. The scale bar represents the substitution percentage. *Nitzschia communis* was used as the outgroup. **b** Dendrogram based on the nuclear rDNA spacers’ sequence (ITS-1, 5.8 S gene and ITS-2). Bootstrap values are given at the nodes. The scale bar represents the substitution percentage. *Stichococcus bacillaris* was used as the outgroup. **c** Dendrogram based on the 18S rRNA gene sequence. Bootstrap values are given at the nodes. The scale bar represents the substitution percentage. *Ankistrodesmus stipitatus* was used as the outgroup. GenBank accession numbers follow the species name in parentheses

A fragment of the 18S rRNA gene and the 3′-end of the *rbc*L gene segment (*rbc*L-3′P) were used to identify the Strain WT4 and Strain WT7 diatom isolates. The *rbc*L-3P and *SSU* rDNA regions were sequenced and compared with sequences already available in GenBank database. Phylogenetic analyses based on *SSU* rDNA and *rbc*L-3P sequences revealed that Strain WT4 and Strain WT7 isolates were members of the Bacillariophyceae class (pennate diatoms) and were grouped within the Thalassiophysales and Bacillariales orders, respectively (Fig. 1a, b). The *SSU* rDNA region and the *rbc*L-3P protein sequences revealed that Strain WT4 and Strain WT7 isolates were related to the *Amphora subtropica* [55] and *Nitzschia draveillensis* [56] species, respectively. These results demonstrated that these isolates (Strain WT4 and Strain WT7) belong to the Catenulaceae and Bacillariaceae families and were closely related to the genus *Amorpha and Nitzschia,* respectively. The Strain WT3 isolate was identified based on the 18S rRNA sequence and both the internal transcribed spacer regions combined with the 5.8S gene (ITS-1 + 5.8S + ITS-2). PCR amplification and subsequent DNA sequencing allowed the determination of approximately 1800 bp of the 18S rRNA gene. Strain WT3 displayed the highest 18S rRNA sequence relatedness with the green alga *Tetraselmis striata* (99% similarity) [57]. Strain WT3 was grouped among the Chlorodendrophyceae class and Chlorophyta phylum (Fig. 2a). The sequence of the nuclear rDNA spacers confirmed that the Strain WT3 isolate was closely related to *Tetraselmis striata* (Fig. 2b). The 18S rRNA gene sequence from the Strain WT1 isolate presented a 99% similarity with *Chlorella* sp. Strain SAG 211-18 [58] and *Chlorella* sp. strain KAS012. This strain was placed among the Parachlorella-clade in the Trebouxiophyceae class and Chlorophyta phylum (Fig. 2a). As previously reported [26], phylogenetic analyses of the 18S rRNA gene sequences placed StrainWT6 in the Scenedesmaceae (Sphaeropleales, Chlorophyceae) family (Fig. 2c). This family has a monophyletic lineage within the Sphaeropleales order consisting of autosporic green algae from the genus *Scenedesmus* and its relatives. The 18S rRNA gene sequence from Strain WT6 showed ≥ 99.9% similarity with *Scenedesmus* (*Acutodesmus*) *rubescens* CCAP 232/1 [59], *Scenedesmus* (*Acutodesmus*) *dissociatus* UTEX 1537 [60], *Scenedesmus* (*Acutodesmus*) *littoralis* [61]*, Scenedesmus* (*Acutodesmus*) *distendus* Hegewald 1975-267 [60] and *Scenedesmus deserticola* BCP-EM2-VF3 [62].

### Performance of native microalgae strains

**Fig. 3:**
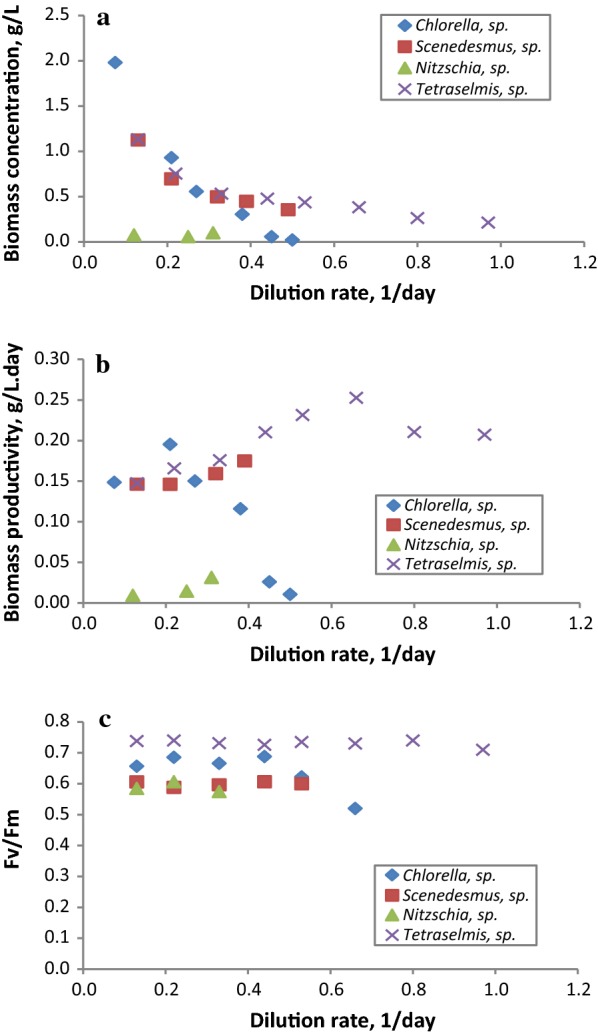
Changes in biomass concentration (**a**), biomass productivity (**b**) and photosynthetic efficiency (**c**) of *Tetraselmis* sp., *Chlorella* sp., *Nitzschia* sp. and *Scenedesmus* sp. once steady state of the cultures was achieved as a function of the dilution rate applied

As a contribution to the survey of microalgae strains which are native to the south-western Mediterranean, the five different newly isolated microalgae strains from north-eastern Tunisia were evaluated in semi-continuous mode at different dilution rates to determine the optimal dilution rate for each strain and to select the best performing strain that could be used in further experiments. Experiments were performed in semi-continuous mode at dilution rates ranging from 0.08 to 0.90 1/day. The diatom *Amphora* sp. exhibited a very low biomass concentration during the first batch phase as well as operational cultivation difficulties such as biofouling; hence, it was not cultivated further in semi-continuous mode. The influence of the dilution rate on biomass concentration and productivity for each strain of the four isolates that grew adequately is displayed in Fig. 3a, b, respectively. For all the strains, the results demonstrated a typical trend of light-limited culture, where the biomass concentration decreased as the dilution rate increased; with a maximum biomass concentration of 2.00 g/L being measured (Fig. 3a). The maximum biomass productivity (0.25 g/L day) was obtained with *Tetraselmis* sp. at 0.65 1/day, while the lowest (0.01 g/L day) was with *Nitzschia* sp. The highest biomass productivity determined in this study (0.25 g/L day) was lower than the 0.87 and 0.76 g AFDW/L day obtained from *S. obliquus* and *C. sorokiniana* DEO1412, respectively—cultivated in batch mode for 200 h [63], and the 0.47 g/L day yielded by *C. pyrenoidosa* in 1-L flask laboratory cultures after 16 days of incubation [64]. However, the productivity obtained is comparable to previously reported values; for instance, San Pedro et al. [25] obtained a biomass productivity of 0.23 g/L day from *Tetraselmis suecica* cultivated semi-continuously at 0.47 1/day. Nonetheless, the productivity reported here is higher than the 47.71, 39.85 and 31.55 mg/L day reported from 15-day batch-cultivated *Chlorella ellipsoidea* in 2-L, 20-L (indoor) and 200-L (outdoor) bubble-column bioreactors, respectively [65].

The very low productivity obtained from the continuous cultivation of the *Nitzschia* sp. strain could be attributable to an inadequate culture medium or unsuitable cultivation conditions. *Nitzschia* sp. is a benthic species that tends to grow adhered to solid substrates, and when cultivated in suspension tends to form lumps. In addition, temperature and pH greatly affect the growth of this microalga [25, 64, 66, 67]. In fact, microscopic observations of this strain carried out during the experiment revealed the presence of cell agglomerates as well as cell morphology alterations. Further experiments focusing on culture condition optimization should be carried out to enhance the biomass productivity of this diatom strain. The maximum photosynthetic activity of PSII for the different strains was measured daily as an indicator of the cells’ physiological status and the steady-state data are presented in Fig. 3c. Of all the dilution rates applied, the Fv/Fm ratio for *Tetraselmis* sp. was the highest compared to the other strains, and remained constant in the 0.71–0.74 range, reflecting the high photosynthetic efficiency of this strain. Similarly, the marine species *Chlorella* sp. presented constant photosynthetic activity (0.69–0.62) and dropped to 0.52 at the washing-out dilution rate, suggesting that the decrease in biomass concentration at that dilution rate (0.50 1/day) was probably associated with high light intensity leading to the photoinhibition of the cells. The Fv/Fm ratio of the freshwater species *Scenedesmus* sp. did not vary significantly at any of the dilution rates and were in the 0.61–0.59 range whilst *Nitzschia* sp. expressed low Fv/Fm ratio values—as low as 0.57—which is in agreement with the biomass productivity obtained, and confirms the hypothesis that this strain’s culture was subject to stress. Nevertheless, the values reported here are similar to those found in previous studies [26, 67, 68].

### Effect of the dilution rate on the biochemical composition of the biomass

**Fig. 4:**
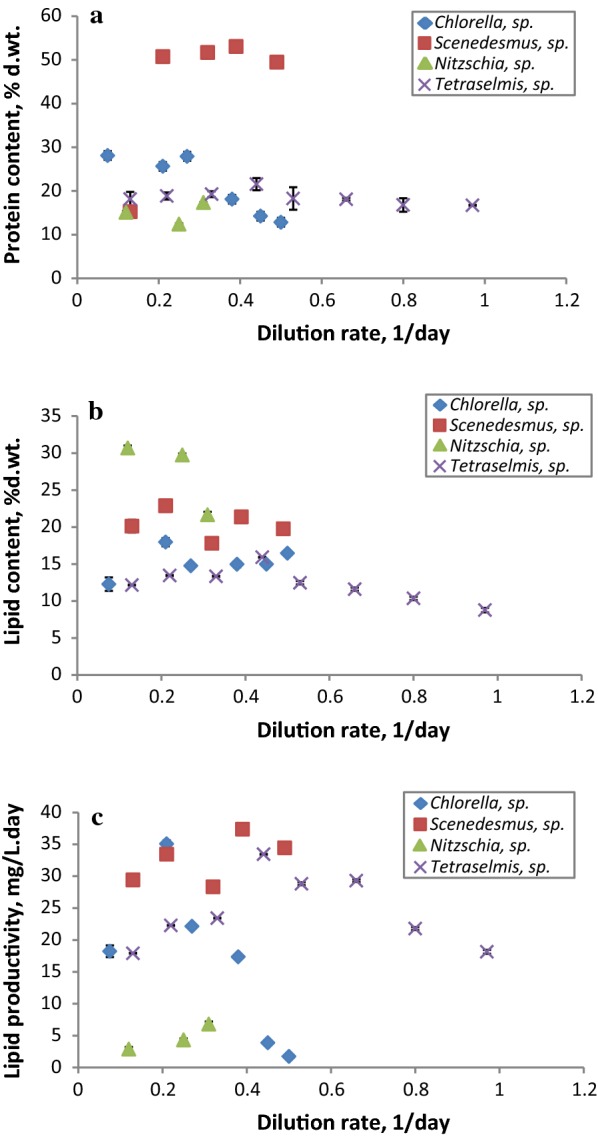
Variations in protein (**a**) and lipid (**b**) contents, and lipid productivity (**c**) of *Tetraselmis* sp., *Chlorella* sp., *Nitzschia* sp. and *Scenedesmus* sp. at steady state, as a function of the dilution rate applied

The biochemical composition of each native microalgae strain was evaluated once steady state was achieved (washing the reactor volume three times). Results shown in Fig. 4a reveal that the protein content is strain dependent and varied from one genus to another. *Scenedemus* sp. presented the largest amount of proteins, increasing from 15.30% d.wt. at 0.10 1/day, to remain constant at all the higher dilution rates (ranging from 53 to 49.50% d.wt.). This would make it potentially interesting as a feedstock for protein-based food processes, following oil extraction, particularly for aquaculture species such as shrimps, molluscs and fish [69, 70]. Similarly, the dilution rate seemed to have no effect on the protein content of *Tetraselmis* sp., which ranged between 16.70 and 19.30% d.wt., although a slight increase (up to 21.60% d.wt.) was noticed at 0.40 1/day. The values reported here were lower than those obtained by Khatoon et al. [71], who cultivated *Tetraselmis* sp. in batch mode at different salinities, yet were comparable to those reported by Kim et al. [72] for *Tetraselmis* sp. KCTC 12236BP after batch mode cultivation in an aerated photobioreactor. As for *Chlorella* sp., the protein content remained constant (25.60–28.10% d.wt.) at the first three dilution rates, then continuously declined as the dilution rate increased, dropping to 12.80% d.wt. at 0.50 1/day. An approximate range of values was observed by Wu and Miao [64], who cultivated *C. pyrenoidosa* indoors in batch mode at different nitrate concentrations. A different trend was observed for *Nitzschia* sp., whereby the protein content increased as the dilution rate increased from 15.10 to 20% d.wt., with a slight decrease at 0.25 1/day. This trend is in accordance with the data found by San Pedro et al. [25] using *Nannochloropsis gaditana*.

Regarding lipid content and productivity, the variation as a function of the dilution rate was studied for the different isolates and the data are shown in Fig. 4b, c. As previously pointed out [23, 68], the crucial criterion in the selection of potential candidates for biofuel production is the lipid productivity rather than the lipid content. For all the isolates, no specific trend was observed relating to the dilution rate applied. For the marine isolate *Chlorella* sp., the total lipid content ranged from 12.30 to 17.90% d.wt. and the lipid productivity was in the 1.70–35.10 mg/L day range—the maximum (17.90%, 35.10 mg/L day) was recorded at 0.20 1/day. A similar lipid content range was determined by Selvarajan et al. [73], who cultivated five microalgae strains of the *Chlorella* genus in batch mode for 21 days, whereas Song et al. [23]. determined a lower lipid productivity (7.96 mg/L day) than that reported in this study for the same genus strain. The lipids content of *Scenedesmus* sp. ranged from 17.80 to 22.90% d.wt. and the lipid productivities were in the range of 28.30–37.40 mg/L day, with the lipid content peaking at 0.20 1/day. A comparable lipid content range was obtained by Yin-Hu et al. [74], who cultivated *Scenedesmus* sp. under phosphorus-starvation and -repletion conditions in batch mode. Nonetheless, the lipid productivities reported here were higher than those determined for *Scenedesmus* sp. in previous studies [23, 75, 76]. *Nitzschia* sp. demonstrated a notable capability for accumulating lipids (20.60–30.70% d.wt.), which decreased as the dilution rate increased. However, the lipid productivity was only 2.90–6.80 mg/L day due to the low biomass productivity. A similar result was observed by Song et al. [23] for diatom strains. *Tetraselmis* sp. exhibited lipid productivities ranging from 17.90 to 33.40 mg/L day, whilst the lipid content was in the 8.80–15.90% d.wt. range, where it continuously decreased at dilution rates higher than 0.40 1/day. This lipid range was lower than that previously reported for the same genus, *Tetraselmis* [71, 72]. The differences in the biochemical composition results for the various isolates from the same phylum and genus demonstrate that they are not only caused by differences in culture conditions but also because the biochemical composition of the microalgae strain is an intrinsic characteristic and species specific. In the light of the results obtained, the marine isolate *Chlorella* sp. demonstrated the best compromise of lipid and biomass productivity at 0.20 1/day; therefore, this microalgae strain was selected for further experiments.

**Fig. 5:**
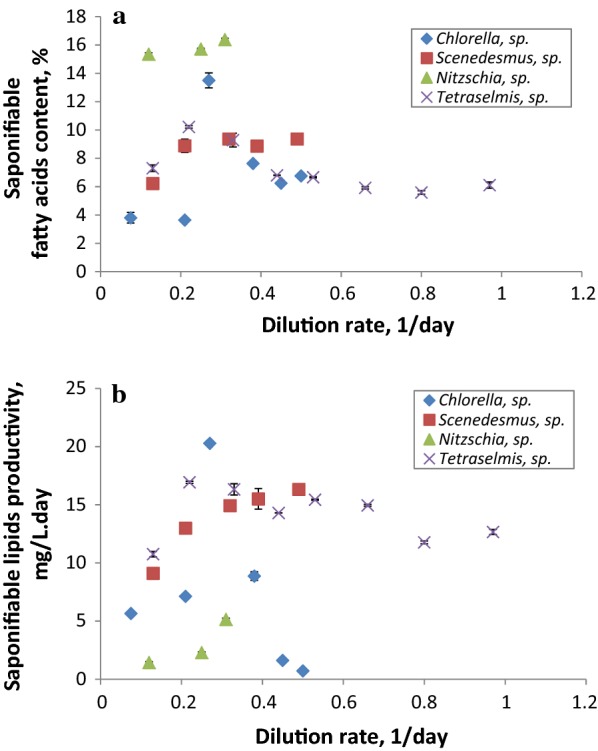
Variations in fatty acids content (**a**) and saponifiable lipids productivity (**b**) of *Tetraselmis* sp., *Chlorella* sp., *Nitzschia* sp. and *Scenedesmus* sp. at steady state, as a function of the dilution rate applied

Another critical criterion for the selection of potential microalgae candidates for biodiesel production (i.e., the direct transesterification of the biomass lipid fraction) is the qualitative fatty acid composition and the saponifiable lipids productivity (i.e., the fatty acid methyl esters [FAME] productivity). In fact, the fatty acid profile and structure significantly influence and dictate the physical and chemical properties of the biodiesel produced, including the octane number, viscosity, cold flow, oxidative stability and lubricity [68, 77]. The saponifiable fatty acid content and profile (Fig. 5a and Table 2, respectively) varied widely amongst the different isolates with no specific trend being observed in relation to the distinct dilution rates applied. It is worth mentioning that the dilution rate is not the only variable impacting on lipid content and lipid productivity in continuous cultures. In fact, the relationship between the lipid productivity, the dilution rate, the medium composition and the average irradiance in microalgae cultures has been previously reported [78]. Besides, the culture growth status, i.e., the average irradiance within the reactor, influences the fatty acid profile and content, because the fatty acid content is a function of the light availability inside the culture and the extent of photolimitation–photoinhibition phenomena [78]. Considering SFA and MUFA in combination, *Tetraselmis* sp. presented a range of 33.40–53.90% of total saponifiable fatty acids—comparatively higher than the other two Chlorophyte isolates. The highest saturated and monosaturated FAME content, however, was observed for the diatom *Nitzschia* sp. (81.40%). As for FAME productivity, the highest value (20 mg/L day) was recorded for *Chlorella* sp. at 0.30 1/day followed by *Tetraselmis* sp. (16.90 mg/L day) at 0.20 1/day and then *Scenedesmus* sp. (16.30 mg/L day) at 0.50 1/day (Fig. 5b). In a study conducted by San Pedro et al. [24], the selected strain for biodiesel production showed higher productivity of accumulated fatty acids up to 51 mg/L day comparing to the one here determined. Similarly, these reported results are far from those obtained by Münkel et al. [79] who cultivated the freshwater strain *Chlorella vulgaris* in batch mode achieving a fatty acid productivity of 0.39 g/L day. However, it is worth noting that these authors carried out a two-stage culture strategy where the second stage is nitrate or/and phosphate-depleted phase to enhance lipid accumulation; whereas in comparison with the present work, the cultivation was performed under sufficient nutrients conditions.

**Table 2:** Summary of fatty acid compositional profiles at steady state for algal lipids from the different isolates cultivated in semi-continuous mode at different dilution rates

	*Chlorella* sp.						*Scenedesmus* sp.				
D, 1/day	0.08	0.20	0.30	0.38	0.45	0.50	0.10	0.20	0.30	0.40	0.50
14:0	–	–	–	–	–	–	–	–	–	–	–
16:0	–	–	–	17.40	15.40	14.80	–	16.40	16.50	16.30	16.00
16:1n7	33.10	16.60	14.50	2.30	–	–	18.80	2.90	2.60	2.60	2.70
16:2n4	6.30	12.90	5.30	4.40	4.20	4.30	3.20	2.30	–	3.00	–
16:3n4	5.00	6.90	10.00	9.50	11.20	10.70	2.90	–	–	–	–
16:4n1	–	–	3.70	2.40	–	–	9.10	11.80	13.50	13.70	13.80
18:0	1.80	–	3.50	–	2.80	3.20	–	–	7.10	6.80	–
18:1n9	8.40	–	2.80	6.90	2.40	2.80	11.70	7.80	–	–	6.90
18:1n7	–	–	–	–	–	–	–	–	–	–	–
18:2n6	–	–	–	14.20	13.40	12.90	–	14.10	11.30	9.70	8.40
18:3n3	9.30	13.20	–	28.10	30.00	28.30	16.20	24.50	28.10	29.90	31.90
18:4n3	16.40	19.50	–	–	–	–	20.20	1.80	1.80	1.80	–
20:4n6	–	–	–	–	–	–	–	–	–	–	–
20:1n9	3.00	3.00	–	2.20	3.20	3.20	–	–	–	–	–
22:5n3	–	–	–	–	–	–	–	–	–	–	–
SFA	1.80	–	3.50	17.40	18.20	18.00	–	16.40	23.50	23.10	16.00
MUFA	44.50	19.60	19.40	11.40	5.60	3.20	30.50	10.70	2.60	2.60	9.60
PUFA	37.00	52.50	57.50	58.70	58.90	56.30	51.60	54.60	54.80	58.10	54.10

	*Nitzschia* sp.			*Tetraselmis* sp.							
D, 1/day	0.10	0.20	0.30	0.10	0.20	0.30	0.40	0.50	0.60	0.80	0.90
14:0	6.60	7.60	6.30	–	–	–	–	–	–	–	–
16:0	23.70	25.20	20.80	14.90	14.60	14.80	16.00	11.40	17.00	17.90	18.00
16:1n7	47.70	43.60	34.40	3.20	3.00	2.30	2.40	2.30	1.90	1.70	1.40
16:2n4	1.80	2.20	4.20	8.00	8.00	7.30	6.00	5.60	5.00	4.50	4.00
16:3n4	1.60	2.40	5.10	0.50	5.30	6.40	8.80	8.90	10.20	11.40	10.30
16:4n1	–	–	–	6.40	12.40	11.70	11.50	11.00	9.80	10.10	10.80
18:0	1.40	1.50	–	12.70	2.30	2.10	0.90	13.90	12.20	10.60	9.80
18:1n9	1.70	1.60	1.80	2.50	17.90	16.40	14.10	14.70	16.50	17.50	20.20
18:1n7	–	–	–	–	13.00	14.50	13.30	–	–	–	–
18:2n6	–	–	–	17.00	–	–	–	2.80	–	–	–
18:3n3	8.00	7.80	19.00	10.90	0.70	0.90	1.60	1.60	2.00	2.30	2.40
18:4n3	–	–	–	0.80	0.90	0.90	1.20	1.20	–	1.40	1.60
20:4n6	–	–	–	2.80	1.00	1.20	1.70	1.90	2.30	2.80	3.10
20:1n9	–	–	–	–	3.00	3.10	2.60	2.40	2.20	2.20	1.80
22:5n3	7.70	7.90	13.00	–	–	–	–	–	–	–	–
SFA	31.80	34.40	27.00	27.60	16.90	16.90	16.90	25.30	29.30	28.50	27.80
MUFA	49.60	45.20	36.20	5.80	37.00	36.40	32.40	19.40	20.60	21.40	23.50
PUFA	11.00	12.50	22.40	46.50	28.30	28.40	31.00	33.20	29.40	32.60	32.30

Data are mean value of two repetitions

SFA: Saturated fatty acids; MUFA: mono unsaturated fatty acids; PUFA: poly unsaturated fatty acids

Palmitic and palmitoleic acids were the predominant fatty acids in most of the algal isolate extracts followed by C18:3n3, C18:1n9, C16:4n1 and C16:3n4 (Table 2). Similar classes of fatty acids were previously reported [80] with some differences in content that might be attributable to the diversity of microalgae strains studied and the culture conditions. The Chlorophyceae *Scenedesmus* sp. and the Trebouxiophyceae *Chlorella* sp. contained high C18:3n3 contents in the 16.20–31.90% range and 9.30–30% of total fatty acids, respectively. The highest amount of C16:1n7 was attributed to the diatom *Nitzschia* sp. (47.70% of total fatty acids at 0.10 1/day), whereas a considerable amount of C18:1 (2.50–30.90% of total fatty acids) was obtained from *Tetraselmis* sp. It is worth noting that, of all the different isolates, only *Nitzschia* sp. presented broad classes of fatty acids from lighter species such as C:14 (6.30–7.60% of total fatty acids) to heavier species like C22:5 (7.70–13% of total fatty acids). Comparing these FAME profiles with those of common and current worldwide biofuel feedstocks [77], the Trebouxiophyceae *Chlorella* sp. and the freshwater *Scenedesmus* sp. presented similar fatty acid profiles at high dilution rates to camelina, characterized by the high C18:3n3 content. Whereas, the flagellate *Tetraselmis* sp. showed (at almost all the dilution rates tested) a fatty acid composition more similar to tallow [77], marked by an abundance of C16:0 and C18:1 as well as a considerable C18:0 content (Table 2).

In addition, a biodiesel quality estimation was conducted for the different isolates. In this study, the most important fuel properties frequently reported in the literature for the assessment of FAME suitability as a fuel (Table 3)—kinematic viscosity, specific gravity, cloud point (CP), cetane number (CN), iodine value (IV) and high heating value (HHV)—were empirically determined using predictive models based on fatty acid composition [77]. The estimated values of CN, CP, HHV, specific gravity and kinematic viscosity for the different FAMEs derived from the isolates complied with almost all the common quality specifications, in accordance with standards ASTM D6751 in the US and EN 14214 in Europe. Furthermore, they are within the value ranges obtained from the widely used biodiesel feedstock (Table 3). The European standard is more stringent, designating a minimum CN value of 51; whereas, the US standard requires a minimum of 47. The CN is a measure of ignition quality in a diesel engine [68, 77]. The results showed a range of 48.80–58.50 for the different strains, in compliance with the standards requirements. The iodine value (IV) is a measure of unsaturation, and thus reflects a biodiesel’s oxidative stability. ASTM D6751 does not designate any IV specification whereas EN 14214 includes a maximum of 120 g I_2_/100 g FAME. *Tetraselmis* sp. and *Nitzschia* sp. exhibited an IV range of 60.90–67.00 and 99.00–112.30 g I_2_/100 g FAME, respectively. This is lower than the prescribed limits, compared to the most common vegetable oils used for biodiesel production and lower than camelina (152.80 g I_2_/100 g FAME), soy (125.50 g I_2_/100 g FAME) and sunflower (128.70 g I_2_/100 g FAME) oils (Table 3) [77]. The kinematic viscosity is a measure of a biodiesel’s flow resistance and this increases as the saturation increases, leading to poor combustion, high emissions and oil dilution [77]. The results presented in Table 3 showed that kinematic viscosity values varied between 3.90 and 4.80 mm^2^/s, which falls within the specifications range. The diatom *Nitzschia* sp. demonstrated the highest kinematic viscosity values, of 4.80 mm^2^/s, probably due to its high SFA and MUFA contents (up to 81.30% of total fatty acids).

**Table 3:** Comparison of estimated biodiesel properties of algal lipids from the different isolates as a function of the dilution rate along with the US and European specifications (B100), and those of common vegetable oils

	*Chlorella* sp.						*Scenedesmus* sp.					*Nitzschia* sp.		
D, 1/day	0.08	0.20	0.30	0.38	0.45	0.50	0.10	0.20	0.30	0.40	0.50	0.10	0.20	0.30
KV, at °C mm^2^/s	4.20	4.00	3.90	4.10	4.20	4.20	3.90	4.10	4.10	4.00	4.00	4.80	4.80	4.70
Specific gravity, Kg/L	0.88	0.88	0.88	0.88	0.88	0.88	0.88	0.88	0.88	0.88	0.88	0.87	0.87	0.87
CP, °C	− 2.10	− 4.60	− 8.00	− 2.90	− 2.00	− 1.00	− 8.20	− 2.90	− 2.80	− 4.00	− 3.70	11.20	11.30	10.20
CN, min	51.80	50.60	48.90	51.40	51.90	52.40	48.80	51.40	51.50	50.90	51.00	58.50	58.50	58.00
IV, g I_2_/100 g	135.80	149.40	169.00	140.20	135.20	130.00	170.00	140.50	139.80	146.40	144.70	61.50	60.90	67.00
HHV, MJ/Kg	41.40	41.80	42.20	41.50	41.40	41.30	42.20	41.50	41.50	41.70	41.60	39.70	39.70	39.80

	*Tetraselmis* sp.								ASTM D6751-08	EN 14214	Camelina	Sunflower	Soy
D, 1/day	0.10	0.20	0.30	0.40	0.50	0.60	0.80	0.90					
KV, at °C mm^2^/s	4.40	4.40	4.40	4.40	4.40	4.50	4.40	4.40	1.9–6.0	3.5–5	3.8	4.42	4.26
Specific gravity, Kg/L	0.87	0.87	0.87	0.87	0.87	0.87	0.87	0.87	–	0.85–0.9	0.88	0.87	0.88
CP, °C	2.60	2.80	2.80	2.10	3.30	4.50	2.70	2.40	–	–	3	2	–
CN, min	54.20	54.30	54.30	53.90	54.60	55.10	54.30	54.00	47	51	50.4	51.1	51.3
IV, g I_2_/100 g	109.50	108.20	108.00	112.30	105.30	99.0	108.80	110.90	–	120	152.8	128.7	125.5
HHV, MJ/Kg	40.80	40.80	40.80	40.90	40.70	40.60	40.80	40.80	–	–	45.2	40.6	39.7

Data are mean value of two repetitions

KV: Kinematic viscosity; CP: cloud point; CN: cetane number; IV: iodine value; HHV: high heating value

In this study, the estimated specific gravity (also referred to as fuel density), which affects engine performance [77], varied between 0.87 and 0.88 kg/L. This is in accordance with the globally accepted standard EN 14214. In contrast, the US and European fuel standards do not state any specification regarding the CP. This property, related to a biodiesel’s low-temperature performance, increases along with an increase in the presence of long-chain SFA resulting in poor cold flow properties [77]. The estimated CP value results for biodiesel from the isolates revealed a wide range, from − 8.20 to 11.30 °C, where the highest CP was determined for the diatom *Nitzschia* sp., indicating poor low-temperature performance. No specifications include HHV values; however, the values determined for the different isolates in the present study (39.70–42.20 MJ/kg) were within the range of common vegetable oil biodiesels and previously reported values from microalgae [73, 77]. Sun et al. [81]. studied the properties of biodiesel from nine *Chlorella* strains. The authors found higher CP values comparing to those here reported; whereas lower IV values were determined. Also, Miao et al. [64] reported the biodiesel properties of *Scenedesmus obliquus* and *Chlorella pyrenoidosa* and showed that they meet several of ASTM specifications. In these both studies, similar range of CN was recorded comparing to that determined in the present work.

### Influence of outdoor conditions on the performance of the selected microalgae strain WT1 *Chlorella* sp.

**Fig. 6:**
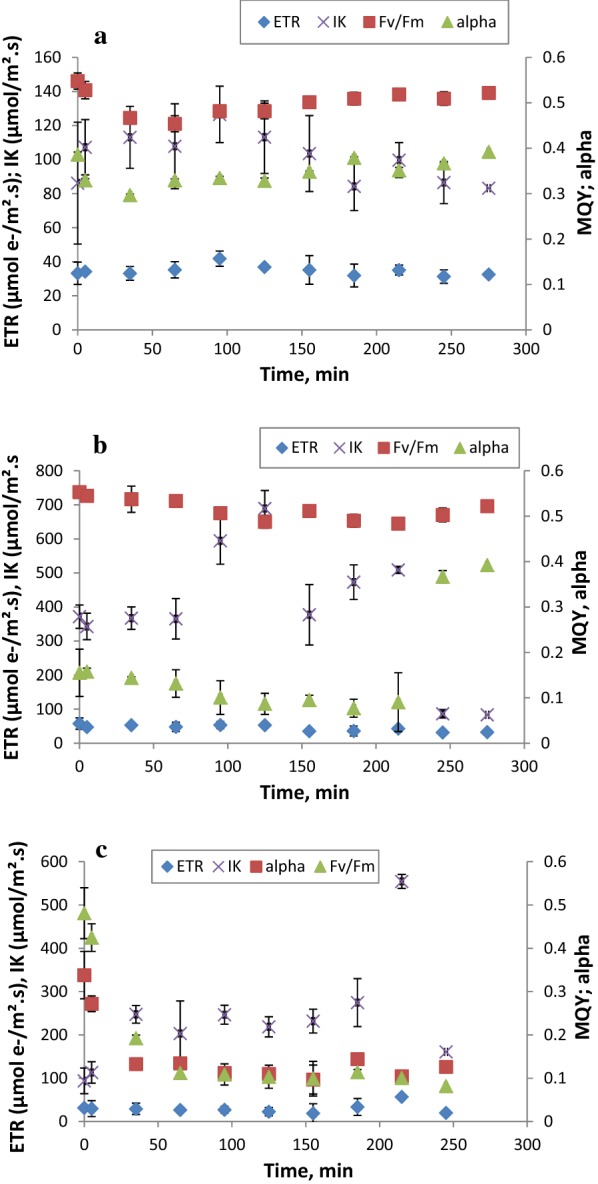
Effect of temperature at 38 °C (**a**), at 10 °C (**b**) and high irradiance (1600 µmol/m^2^ s) (**c**) on different photosynthetic parameters of the WT1 *Chlorella* sp. strain as a function of time

The effect of temperature and light prevailing outdoor conditions on the performance of the selected microalgae strain *Chlorella* sp. was studied. The effect of high and low temperature (38 °C and 10 °C) is shown in Fig. 6a, b. Multivariate analyses based on Pillai’s Trace test showed that both temperature, at the two levels (38 °C and 10 °C), and the length of exposure to the tested temperature (time), had a highly significant effect on the photosynthetic parameters (*p* < 0.001). The interaction between these factors also showed a synergy (Pillai’s trace value = 1.314; *F* = 2.516; *p* < 0.001). As can be observed in Fig. 6a, the Fv/Fm decreased due to high temperature (38 °C) and recovery came about only after 155 min. In addition, the curve of light saturation intensity (Ik) exhibited a notable decrease starting at 125 min. On the other hand, Fig. 6b displayed a different low-temperature-effect pattern (10 °C), where alpha constantly dropped from 0.15 to 0.09 at 215 min and then suddenly increased. Furthermore, the saturation irradiance showed a variable trend during the experiment time. Total Fv/Fm variability was mainly governed by the exposure time (74%); whereas, the temperature effect accounted for 90% and 98% on Ik and alpha, respectively. However, temperature and time exposure almost equally controlled ETR (58% and 42%, respectively).

Regarding the light effect (1600 µmol/m^2^ s), the results for the photosynthetic parameter variations as a function of time are displayed in Fig. 6c. They reveal that highlight intensity had a major impact on ETR and alpha (*p* < 0.001) while influencing Ik less (*p* < 0.005). The exposure time to high irradiance greatly affected ETR and Fv/Fm, according to multivariate test analyses using Pillai’s Trace test. Similarly, the interaction effect between these two factors was found to be highly significant (Pillai’s Trace value = 1.769; *F* = 2.677; *p* < 0.001). Additionally, a highly significant effect of this synergy was noted on ETR and Fv/Fm (*p* < 0.001). The figure shows that both alpha and Fv/Fm sharply decreased after 35 min and 5 min, respectively, of exposure to high irradiance. Similar results were found by [82], where Fv/Fm reduced after 5 min of exposure to high irradiance. In fact, some studies have reported on photo-protective mechanisms that aim to protect photosynthetic reaction centers from over excitation caused by high irradiance or temperature variations. The determination for the corrected total sum of square partitioning indicated that ETR and Fv/Fm were 81% and 83% controlled by high irradiance, respectively, while alpha was controlled by the length of exposure to high light intensity (68%).

Consequently, after evaluating all these results, the marine strain *Chlorella* sp. was selected as the most promising candidate for renewable fuel production due to its considerable biomass and lipid productivities (0.20 g/L day and 35.10 mg/L day, respectively, at 0.20 1/day), the suitability of its fatty acid profile with respect to biofuel properties, and its important FAME productivity. The investigation into this selected marine isolate’s photosynthetic characteristics showed accordance with the results, demonstrating that the *Chlorella* sp. strain is sufficiently tolerant and robust when faced with severe outdoor conditions.

## Conclusions

Five native Tunisian microalgae strains were isolated and identified. These were *Scenedesmus* sp., *Tetraselmis* sp., *Chlorella* sp., *Amphora* sp. and *Nitzschia* sp. Four isolates were successfully cultivated in semi-continuous mode at different dilution rates under laboratory conditions, which simulated outdoor conditions, in order to evaluate their potential for biofuel production. *Chlorella* sp. demonstrated the best performance—at 0.20 1/day, it achieved a lipid productivity of 35.10 mg/L day; while at 0.3 1/day, it achieved a FAME productivity of 20.30 mg/L day. Furthermore, when imposing severe outdoor conditions, the selected strain showed acceptable tolerance, with a photosynthetic parameters assessment using chlorophyll-fluorescence analysis revealing prompt culture recovery.

## Data Availability

The data sets used and/or analyzed during the current study are available from the corresponding author on reasonable request.
